# Serum and Urinary Uromodulin in Dogs with Early Chronic Kidney Disease vs. Healthy Canine Population

**DOI:** 10.3390/ani14142099

**Published:** 2024-07-18

**Authors:** Nikola Marečáková, Jana Kačírová, Csilla Tóthová, Aladár Maďari, Marián Maďar, Mária Kuricová, Slavomír Horňák

**Affiliations:** 1 Small Animal Clinic, University Veterinary Hospital, University of Veterinary Medicine and Pharmacy in Kosice, 041 81 Kosice, Slovakia; n.marecakova@gmail.com (N.M.); maria.kuricova@uvlf.sk (M.K.); slavomir.hornak@uvlf.sk (S.H.); 2 Institute of Plant Genetics and Biotechnology, Plant Science and Biodiversity Centre, Slovak Academy of Sciences, 949 01 Nitra, Slovakia; 3 Clinic of Ruminants, University Veterinary Hospital, University of Veterinary Medicine and Pharmacy in Kosice, 041 81 Kosice, Slovakia; csilla.tothova@uvlf.sk; 4 Department of Microbiology and Immunology, University of Veterinary Medicine and Pharmacy in Kosice, 041 81 Kosice, Slovakia; marian.madar@uvlf.sk

**Keywords:** Tamm–Horsfall protein, biomarker, tubular marker, urine, renal disease, dog

## Abstract

**Simple Summary:**

Simple Summary: Uromodulin has been known for many years as a normal protein component of urine. However, in recent years, serum as well as urinary uromodulin have been investigated in relation to kidney diseases. Chronic kidney disease (CKD) is a common diagnosis, especially in old dogs, and suitable markers for the early stages of the disease are constantly being investigated. We focused on the effect of age and gender on uromodulin in serum and urine in a healthy population of dogs of the same breed, German shepherd. We compared the results with two groups of other breeds of healthy dogs. Compared with groups of patients in the early stages of CKD, we concluded that urinary uromodulin corrected to creatinine in urine seems to be the most prospective marker.

**Abstract:**

Serum and urinary uromodulin are evaluated as potential biomarkers of kidney disease. The aim of our research was to select a more appropriate form of uromodulin for the diagnosis of early stages of chronic kidney disease (CKD). We also focused on the influence of age and gender in one breed on uromodulin and on the possible interbreed differences. Serum uromodulin had the lowest values in dogs younger than 2 years but no effect of gender, breed, or CKD was observed. Urinary uromodulin indexed to urinary creatinine was significantly reduced in dogs in stage 2 of CKD (*p* = 0.003) in contrast to uromodulin converted to urine specific gravity. Urinary uromodulin with both corrections was significantly lower in Belgian shepherds compared to German shepherds (*p* < 0.0001, *p* = 0.0054) but was not influenced by gender or age. In stage 1 of CKD, urinary uromodulin correlated with kidney disease markers SDMA (*p* = 0.0424, *p* = 0.0214) and UPC (*p* = 0.0050, *p* = 0.0024). Urinary uromodulin appears to be more associated with CKD than serum uromodulin. Further studies with a larger number of patients are needed for the suitability of urinary uromodulin as a marker of early-stage disease.

## 1. Introduction

Uromodulin or Tamm–Horsfall protein is a secretory glycosylphosphatidylinositol-anchored glycoprotein consisting of 616 amino acids that is produced in the epithelial cells lining the thick ascending limb of the loop of Henle, the TAL cells in the kidney, and is released bidirectionally to urine and the interstitial space/circulation [1,2]. Uromodulin in urine was discovered several decades ago but came into the spotlight after the description of mutations in its encoding gene *UMOD* causing renal Mendelian disease (autosomal dominant tubulointerstitial kidney disease). Uromodulin is involved in the regulation of salt reabsorption, cation homeostasis, kidney stone disease, urinary tract infection, systemic inflammation, and stress. Changes in uromodulin levels in urine and serum began to be associated with kidney function disorders, cardiovascular diseases, and hypertension but also with prognosis in chronic kidney disease [3]. 

Uromodulin can be detected in blood serum in monomeric form, while, in urine, it is found at much lower levels as polymeric filaments [4]. Uromodulin in urine and, to a lesser extent, in blood is expected to be a valuable biomarker of renal dysfunction, especially of tubular function. According to the current pathophysiological understanding, uromodulin may not be an indirect marker of glomerular filtration but a direct marker of the number of functional nephrons, renal tissue, or tubular secretion. Tubulointerstitial lesions potentially help to better assess total nephron mass and renal function in contrast to conventional markers of glomerular filtration, which may not yet indicate functional impairment. These findings corroborate studies with decreasing plasma uromodulin in people with predominant glomerulonephritis and in subgroups of patients with glomerular diseases and tubulointerstitial diseases, in which similar results were found [1,5]. In patients with glomerulopathies in the early stage of tubular atrophy and renal fibrosis, serum uromodulin decreased, while estimated glomerular filtration rate (eGFR) values still remain normal [6]. Common markers such as creatinine, BUN, and cystatin C show a hyperbolic correlation with eGFR, while serum uromodulin shows a linear correlation with eGFR [1]. In particular, studies in human medicine have shown that higher urinary uromodulin was associated with a lower risk of decline in eGFR, a lower risk of developing chronic kidney disease (CKD), a lower risk of postoperative acute kidney injury (AKI), and a lower risk of mortality in individuals with older age [2,4,7]. Another study showed that patients who developed CKD during sampling had significantly lower serum uromodulin levels (126.8 ng/mL ± 42.3 ng/mL) than healthy subjects (180.2 ng/mL ± 79.1 ng/mL) in whom CKD was not confirmed at the time of sampling. Serum uromodulin was inversely associated with the development of CKD, even after adjustment for patient age, sex, genotype of the identified polymorphism, hypertension, diabetes status, and eGFR [8]. However, other studies have shown that serum uromodulin is elevated in patients with CKD, attributing increased serum uromodulin levels to significant impairments of renal function in humans [9,10]. Furthermore, serum uromodulin has been confirmed to be the best biomarker for identifying early-stage CKD in human patients and distinguishing between stage 0 and stage 1 patients, where its concentration decreased in serum with disease progression [1].

When studying the entire urinary proteome and metabolome in dogs, the most abundant proteins in urine samples from healthy dogs were recorded as uromodulin, albumin, and arginine esterase. Uromodulin concentrations were significantly lower, while albumin concentrations were significantly higher in urine samples from CKD dogs than in healthy dogs [11]. There is a lack of available studies proving the relationship of uromodulin to CKD in the veterinary field. As the most readily abundant protein excreted in physiologically healthy urine, we believe it could be a good candidate for showing the earliest variations in kidney function.

Several breeds of dog are associated with hereditary CKD, but there is no apparent breed predisposition for nonhereditary CKD. The frequency of breeds in which CKD was confirmed is related to owner preferences in the area where the study was conducted. In general, the disease was most often observed in small breeds of dogs, but the association between these factors has not yet been described [12]. The top 25 dog breeds tested for renal biomarkers of glomerular filtration rate in a US commercial laboratory database (from July 2015 to December 2017) were identified. In the top 10 were the breeds Labrador retriever, Golden retriever, Chihuahua, Yorkshire terrier, Shih tzu, Dachshund, German shepherd dog, Boxer, Beagle, and Jack Russell terrier [13]. The German shepherd is one of the most popular dog breeds in the world and is widely used in guarding, police, military, or guide-dog roles [14]. In Slovakia, the German shepherd is the most popular breed of dog used by the police and the army. Therefore, in this study, we focused on the determination of uromodulin in serum and urine in a healthy German shepherd population and comparison with another working breed (Belgian shepherd) and small breed dogs.

## 2. Materials and Methods

### 2.1. Animal

In this study, we determined the uromodulin level in serum and in urine in a canine population of 98 dogs. All applicable international, national, and institutional guidelines for the care and use of animals were followed. Clinical examinations and sampling were performed with the informed consent of the dog owners.

Dogs were considered healthy or diseased on the basis of history, clinical signs, and the results of routine laboratory tests. Out of 83 healthy dogs, 38 were males (1 castrated) and 45 females (3 spayed). The most common breed was German shepherd (*n* = 71), followed by Belgian shepherd (*n* = 6), Yorkshire terrier (*n* = 1), Maltese (*n* = 1), and small mixed breed (*n* = 4). German shepherds were divided into groups according to age, Belgian shepherds were aged between 0.8 and 6 years, and the other group of small dogs were aged 2 to 9 years (Table 1).

The diagnosis of CKD was based on history, clinical signs, and clinicopathological and imaging results, according to IRIS (2023) [15], in particular, the presence of clinical findings, abdominal imaging results, and persistent pathologic renal proteinuria based on the UPC (UPC > 0.5), assessed and confirmed over a one-month period, or serum creatinine (Crea) concentration or serum symmetric dimethylarginine (SDMA).

The diseased group included 15 dogs affected by naturally occurring CKD, in CKD 1: 7 dogs and in CKD 2: 8 dogs. The 15 CKD dogs were 8 males (1 castrated) and 7 females (1 spayed). There were 2 dogs that were mixed breed and 13 dogs were German shepherds.

### 2.2. Collection and Analysis of Samples

Blood was collected from the *vena cephalica antebrachii* after at least 12 h of fasting period into tubes without anticoagulant. After centrifugation (3500 RPM, 10 min), the serum was subjected to a complete biochemistry profile in the Cobas C 111 (Roche, Switzerland). Serum SDMA level was measured with the Catalyst One (IDEXX Laboratories, Westbrook, ME, USA). Hematology was analyzed in the ProCyte Dx (IDEXX Laboratories, Westbrook, ME, USA). Urine was collected by ultrasound-guided cystocentesis. After routine urinalysis, the urine was centrifugated (2500 RPM, 5 min). Subsequently, the samples were stored at a temperature of 4 °C and examined without freezing and thawing within 72 h. 

Uromodulin was measured in blood serum and in urine with Canine Uromodulin ELISA (BioVendor—Laboratorni medicina a.s., Brno, Czech Republic), a sandwich enzyme immunoassay for the quantitative measurement of canine uromodulin. Standards and samples were incubated in microtitration wells precoated with polyclonal anti-canine uromodulin antibody. After 60 min of incubation followed by washing, biotin-labelled polyclonal anti-canine uromodulin antibody was added and incubated with the captured uromodulin for 60 min. After another washing, the streptavidin–horseradish conjugate was added. After 30 min of incubation and the last washing step, the remaining conjugate was allowed to react with the substrate solution. The reaction was stopped by addition of an acidic solution and the absorbance of the wells was read spectrophotometrically on an automatic photometer for Opsys MR microtiter plates (Dynex Technologies, Chantilly, VA, USA). The absorbance was proportional to the concentration of canine uromodulin. A standard curve was constructed by plotting absorbance values against uromodulin concentrations of standards and concentrations of unknown samples were determined using this standard curve. To avoid diluting effects of urine samples due to variation in water intake, urinary uromodulin concentration was normalized to urine specific gravity (1.025) and urinary uromodulin-to-creatinine ratio (Uurom/1 g Crea) was calculated.

### 2.3. Statistical Analysis

Data processing and statistical analysis were performed using GraphPad Prism 9.4.0 statistical software (GraphPad Software, San Diego, CA, USA). An unpaired *t*-test with Welch’s correction at *p* < 0.05 or a one-way analysis of variance (ANOVA) with an additional Tukey’s test was used to determine statistical differences between healthy dogs and dogs with CKD but also between dogs of different age, breed, and sex. Interactions between the analyzed parameters of healthy dogs and dogs with CKD were evaluated using Pearson’s correlation analysis at *p* < 0.05.

The calculation method for reference interval (RI) and confidence interval (CI) with respect to the sample size, its descriptive statistics, and the normality of the distribution was parametric with a logarithmic transformation. Outliers were identified and removed using the ROUTH method, Q = 1% in GraphPad Prism 9.4.0. Statistical tests for the occurrence of (potential) outliers and for normality were at the 5% significance level, the RI was 95%, and the CI for the limits was 90%.

## 3. Results

A total of 98 dogs were involved in the study. In total, 15 of them were included in the groups of dogs with CKD 1 (*n* = 7) or CKD 2 (*n* = 8). When examining the serum, we focused on conventional parameters such as creatinine, urea, and SDMA and the potential biomarker uromodulin (Table 2). Serum creatinine values were higher than the reference interval in both CKD 1 (102.96 ± 22.38 µmol/L) and CKD 2 (127.75 ± 17.88 µmol/L) dogs. Concentration of urea and SDMA was highest in dogs with CKD 2. Urinary UPC had a similar pattern, with values higher than the reference interval in dogs with CKD 2 (0.27 ± 0.03). Also, urinary creatinine values were the highest in this group of dogs (Table 3).

**Figure 1:**
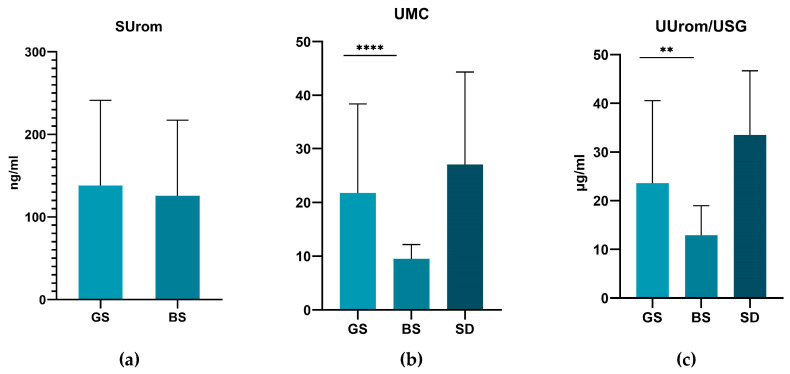
Levels of serum uromodulin (**a**), urinary uromodulin-to-creatinine ratio (**b**), and correction of uromodulin on urinary specific gravity (**c**) according to breed. Values are presented as mean ± SD. The asterisks represent statistical significance: ** *p* ≤ 0.01; **** *p* ≤ 0.001. GS—German shepherds, BS—Belgian shepherds, SD—small dogs.

While serum uromodulin levels were higher in dogs with CKD, urinary uromodulin levels were lower in dogs with CKD. Surprisingly, healthy Belgian shepherd dogs had the lowest values of urinary uromodulin, urinary uromodulin-to-creatinine ratio (UMC), and urinary uromodulin converted to urine specific gravity (UUrom/USG). Between the group of German shepherds and the group of Belgian shepherds, the levels of UMC (*p* < 0.0001) and UUrom/USG (*p* = 0.0054) were significantly lower (Figure 1). We did not observe any significant difference between the group of German shepherds and the group of small breeds. Serum uromodulin was not determined in the group of small breed dogs. 

**Figure 2:**
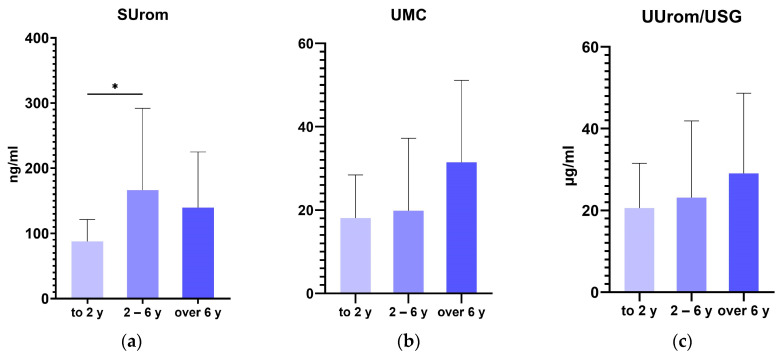
Levels of serum uromodulin (**a**), urinary uromodulin-to-creatinine ratio (**b**), and correction of uromodulin on urinary specific gravity (**c**) according to age. Values are presented as mean ± SD. The asterisk represents statistical significance, * *p* ≤ 0.05. y—years.

Among groups of German shepherd dogs divided by age, the concentration of UMC and UUrom/USG increased with increasing age. There was a statistically significant difference in SUrom values between the group of dogs under 2 years and the group of dogs between 2 and 6 years of age (*p* = 0.0167); in dogs older than 6 years, SUrom values were lower (Figure 2).

**Figure 3:**
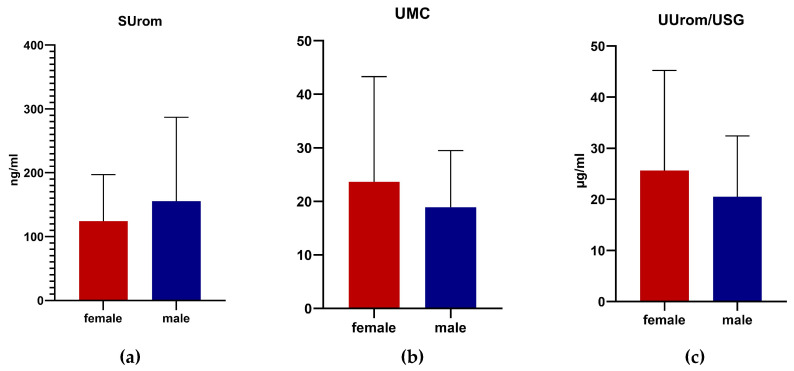
Levels of serum uromodulin (**a**), urinary uromodulin-to-creatinine ratio (**b**), and correction of uromodulin on urinary specific gravity (**c**) according to gender. Values are presented as mean ± SD.

SUrom levels were higher in males, while UMC and UUrom/USG were higher in females. However, no significant differences were demonstrated based on gender in all monitored parameters in German shepherd dogs (Figure 3).

**Figure 4:**
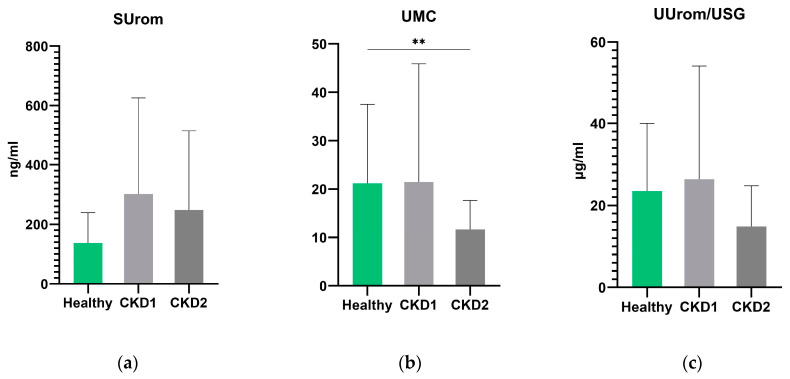
Levels of serum uromodulin (**a**), urinary uromodulin-to-creatinine ratio (**b**), and correction of uromodulin on urinary specific gravity (**c**) in healthy dogs and in dogs with chronic kidney disease stage 1 and chronic kidney disease stage 2. Values are presented as mean ± SD. The asterisks represent a statistical significance, ** *p* ≤ 0.01.

In general, no significant differences were found between groups of dogs with CKD 1 and CKD 2 for all markers examined (Figure 4). UMC was significantly lower in the group of dogs with stage 2 CKD than in the control group of all healthy dogs (*p* = 0.003). UUrom/USG levels were lower in CKD 2 dogs but not significantly (*p* = 0.0521). There were no statistically significant differences in serum uromodulin between the groups of healthy and CKD 2 dogs.

**Figure 5:**
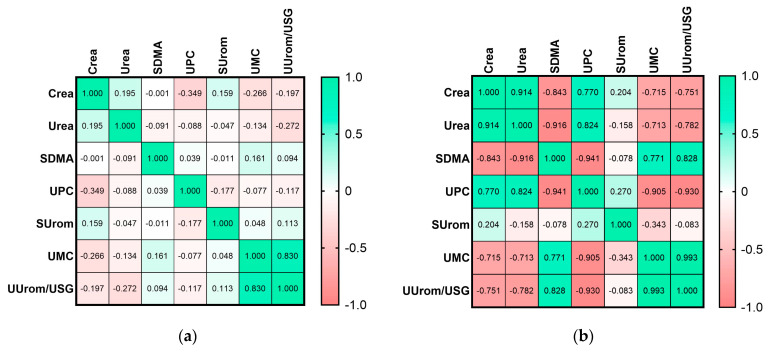

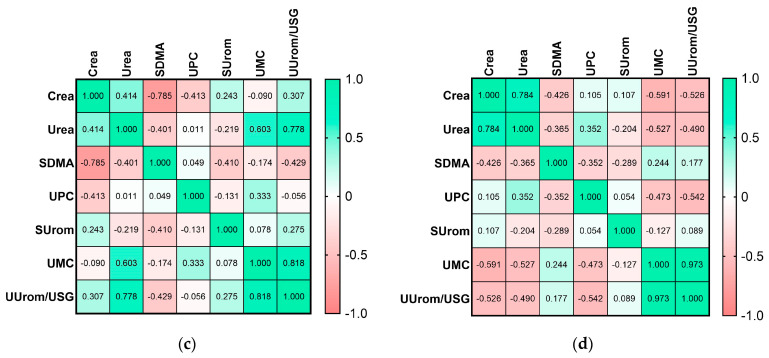
Heatmap of Pearson correlation between the investigated markers and conventional parameters in healthy dogs (**a**), in dogs with chronic kidney disease stage 1 (**b**), chronic kidney disease stage 2 (**c**), and in all dogs with chronic kidney disease (**d**). Crea—serum creatinine, SDMA—symmetric dimethyl arginine, UPC—urinary protein-to-creatinine ratio, SUrom—serum uromodulin, UMC—urinary uromodulin-to-creatinine ratio, UUrom/USG—correction of uromodulin on urinary specific gravity.

Correlations between the investigated markers and conventional parameters (Crea, Urea, SDMA, and UPC) are shown in Figure 5. There was no significant correlation between SUrom and conventional parameters in either healthy dogs or dogs with CKD. Between UMC and Crea, there was a significant correlation in the group of all healthy dogs (r = −0.266, *p* = 0.0217) and in the group of all dogs with CKD (r = −0.591, *p =* 0.0204); a negative correlation with urea was also reported in CKD dogs (r = −0.527, *p* = 0.0437). In group of dogs with CKD 1, we found a correlation between UMC and SDMA (r = 0.771, *p* = 0.0424) and UPC (r = −0.905, *p* = 0.0050). UUrom/USG significantly correlated with urea in all healthy dogs (r = −0.272, *p* = 0.0191) and in the group of dogs with CKD 2 (r = 0.778, *p* = 0.0231). In the group of CKD 1, there was a significant correlation between UUrom/USG and urea (r = −0.782, *p* = 0.0376), UPC (r = −0.930, *p* = 0.0024), and SDMA (r = 0.828, *p* = 0.0214). In the group of all dogs with CKD, we recorded a correlation between UUrom/USG and Crea (r = −0.526, *p =* 0.0438) and UPC (r = −0.542, *p* = 0.037).

For perspective markers, a reference interval was established for all healthy dogs and only for German shepherds. The reference interval (RI) for SUrom in all healthy dogs (*n* = 71) was between 43.65 and 256.80 ng/mL and, only in German shepherds (*n* = 65), the RI was 45.99–245.65 ng/mL. For UMC in all healthy dogs (*n* = 72), the RI was 5.08–52.64 and, only in German shepherds (*n* = 60), the RI was 5.43–51.72. For UUrom/USG in all healthy dogs (*n* = 71), the RI was 6.57–52.22 µg/mL and, only in German shepherds (*n* = 58), the RI was 6.94–46.54 µg/mL.

## 4. Discussion

The aim of the present study was to evaluate uromodulin levels as an early marker of CKD in dogs in the early stages of the disease, CKD 1 and CKD 2. We compared serum uromodulin with urinary uromodulin and converted it with creatinine levels and urine specific gravity. As part of the research, we evaluated the relationship of individual markers and their influence by age, gender, and breed, as well as the body weight of dogs. We further investigated the correlation of uromodulin with common serum markers of kidney disease such as creatinine, urea, and SDMA.

Uromodulin has been studied using both serum and urine concentrations, but urinary uromodulin can be affected by factors such as centrifugation, vortexing, storage conditions, or duration; therefore, serum uromodulin may represent a better and more stable biomarker [16]. However, circulating uromodulin concentrations were much lower than urinary uromodulin concentrations, as found in an earlier study in dogs [11]. Also, in the present study, uromodulin concentrations were lower in serum than in urine. Urinary uromodulin levels remain constant across individuals and indicate the stability of uromodulin excretion in humans; the point concentration of uromodulin and the 24 h concentration of uromodulin were similar. The association between urinary uromodulin and eGFR is positive and linear when eGFR is 90 mL/min per 1.73 m^2^ [17].

The relationship between gender and serum uromodulin was presented by Risch et al. [9]; there were significantly higher values in women but less pronounced than the relationship between uromodulin and CKD. We found higher values of serum uromodulin in males than in females; in contrast, urinary uromodulin was highest in females, but the results were not significant. Urinary excretion of uromodulin is affected by common variants in the *UMOD* gene [18]. The mutation rate is much higher in men than in women because the number of germ cell divisions per generation is significantly higher due to genetics [19]. Common mutations of the *UMOD* gene can reduce urinary levels of uromodulin. There may also be certain hormonal interactions with uromodulin; the kidneys are under the influence of sex hormones. Studies have shown that estrogens have a beneficial role in the progression of some chronic kidney diseases. They have nephroprotective effects [20]. Nanamatsu et al. [21] identified the *UMOD* locus as an estrogen-responsive gene because serum uromodulin tended to decrease in women with increasing age. Another study found that women tended to be in the lowest quartile of uromodulin expression but also tended to be older [7]. Therefore, the higher uromodulin-to-creatinine ratios in women were due to lower urinary creatinine values and not due to higher uromodulin values [17].

The previous study reported a clear negative association between age and urinary uromodulin excretion in humans. However, age was no longer a determinant of uromodulin when 24 h creatinuria was introduced into the model because creatinuria depends on lean body mass and age. Adjustment for creatinine might therefore lead to some form of overcorrection [17]. Our findings did not confirm previous study, as we observed a slight increase in both serum uromodulin and urinary uromodulin with increasing age in a group of healthy dogs; moreover, serum uromodulin was significantly lower in young dogs under 2 years than in dogs between 2 and 6 years of age. 

When comparing individual groups of different breeds and sizes of dogs, we evaluated significant differences in the determination of urinary uromodulin between German shepherd and Belgian shepherd breeds, while we did not notice a difference between German shepherds and small breeds. This could be due to gender representation in the Belgian shepherd group. Although, in our results, the values of urinary uromodulin were slightly lower in males compared to females, the study by Spinella et al. [22] reported significantly higher values of uromodulin in the urine of female working military dogs, which would agree with the predominance of male Belgian shepherds in our work. In humans, it has been shown that there is a correlation between nephron mass and uromodulin excretion. This study was performed on human patients who donated a kidney and found that there was a direct correlation with the amount of uromodulin excreted [23]. With long-term obesity, urinary protein excretion increases and the gradual loss of nephron function worsens over time and exacerbates hypertension. A higher weight can be a cause of CKD; obesity also causes renal vasodilation and glomerular hyperfiltration [24].

A recent human study showed that lower serum uromodulin in patients with CKD was associated with a higher risk of progression to end-stage disease [5]. Similarly, in a recent study in dogs, when comparing control and CKD groups, serum uromodulin concentrations were lower in all dogs with CKD (stages 1–4) compared to controls [25]. In contrast, in our study, we noted increased levels of serum uromodulin in dogs in the early stages of CKD. This is consistent with a human study where there was a trend toward higher serum uromodulin levels in individuals with low GFR. Among other things, they found that the lower the GFR, the lower the urinary excretion of uromodulin. Low urinary uromodulin was also associated with tubular and interstitial atrophic infiltration found in renal biopsies. On the other hand, patients with very low uromodulin in both urine and serum had the highest tubular atrophy scores [26]. Regarding urinary uromodulin, our results agree with previous studies that found low concentrations in dogs with CKD [11,27,28]. Other studies have shown that uromodulin itself may have limitations. A study that evaluated many markers, including uromodulin, for the diagnosis of the preclinical stage of CKD in children did not confirm uromodulin as a useful tool for the diagnosis of early stages of kidney disease [29]. In contrast, the human study by Yazdani et al. [30] showed that the assessment of serum uromodulin together with creatinine or cystatin C allowed a more accurate determination of the risk of mortality in patients with kidney disease. California sea lions excrete a repertoire of proteins in their urine that are close to dogs and humans. Sea lions with leptospirosis also showed a lower level of uromodulin in their urine [31].

Creatinine is an accurate marker of kidney disease and, for this reason, we expected a significant relationship between uromodulin and creatinine. The theory was confirmed for us especially at UMC. A human study evaluating uromodulin secretion as a marker of tubular function showed a positive association between uromodulin and urinary electrolytes, osmolality, creatinine excretion, and urine volume over a 24 h period [17]. As TAL mass is lost and CKD progresses, serum and urinary uromodulin are reduced; moreover, urea drives additional post-translational modifications, such as carbamylation of serum uromodulin, likely leading to loss or alteration of function [32]. A study in dogs also showed that urinary uromodulin was negatively correlated with serum creatinine and UPC [27]. Another study showed that creatinine did not correlate with age, was higher in males than females and in certain breeds of dogs, and showed many similar attributes to the mode of uromodulin secretion [33]. The serum uromodulin concentration showed significant negative correlations with BUN, creatinine, and SDMA in dogs [25]. We recorded a positive correlation in patients with CKD in stage 1 between urinary uromodulin and SDMA and UPC. In a study by Kules [34] focusing on the hemoprotozoal parasite *Babesia canis*, which causes kidney dysfunction in dogs, they used both SDMA and uromodulin as markers of kidney damage. They also found that uromodulin showed no correlation with SDMA in CKD patients. This study shows that, although both are valuable biomarkers for CKD, they show no correlation. This could be due to the decoupling of their biological roles, and the increase in both is due to the dysfunction of completely different biological systems or in different parts of the same system. According to previous mentioned studies in humans and dogs, both serum and urinary uromodulin tend to decrease in CKD. Some cohort studies demonstrate a decrease in serum uromodulin before the onset of azotemia, proteinuria, or an increase in SDMA concentration.

We identified some potential limitations of our study. Despite the finding of significant differences in urinary uromodulin, this result may be influenced by the small number of dogs in the control groups compared to one breed, so more detailed studies focusing on the effect of dog size and weight on urinary uromodulin excretion are needed. Moreover, in the groups of individual breeds of Belgian shepherd dogs and small dogs, the representation of one gender prevailed. In our work, we could not perform a comparison of serum uromodulin of German shepherds and small breeds due to the lack of serum in small dogs at the time of examination of the samples.

## 5. Conclusions

From our results, we conclude that urinary uromodulin is more related to kidney disease than serum uromodulin. Urinary uromodulin converted to creatinine showed significantly lower values in stage 2 CKD compared to healthy dogs, while uromodulin converted to urine specific gravity was not significantly decreased. Although we did not find significant differences in stage 1 CKD, urinary uromodulin correlated with common markers of kidney disease. We conclude that further studies with a larger number of dogs focusing on demonstrating the suitability of urinary uromodulin as a biomarker for early stages of CKD would be beneficial.

## Figures and Tables

**Table 1:** Distribution of dogs within the group of healthy animals.

	Total (*n*)	GS < 2 y	GS 2–6 y	GS > 6 y	BS	SD
Total (*n*)	83	20	36	15	6	6
female	45	12	19	8	1	5
male	38	8	17	7	5	1

GS—German shepherds, BS—Belgian shepherds, SD—small dogs, y—years.

**Table 2:** Serum markers in healthy dogs and dogs with kidney diseases.

	Crea (µmol/L) RI: 46–88	Urea (mmol/L) RI: 3.97–8.05	SDMA (mg/dL) RI: <14	SUrom (ng/mL)
**GS < 2 y**	70.92 ± 16.41	5.29 ± 1.62	9.10 ± 1.80	87.62 ± 33.53
**GS 2–6 y**	84.66 ± 10.90	5.80 ± 1.40	8.22 ± 1.91	166.26 ± 125.48
**GS > 6 y**	78.88 ± 14.07	5.42 ± 1.95	8.92 ± 2.15	139.38 ± 85.36
**BS**	81.83 ± 16.17	5.88 ± 1.68	7.67 ± 3.08	125.81 ± 91.33
**SD**	59.48 ± 11.06	5.94 ± 1.07	8.00 ± 1.79	-
**CKD1**	102.96 ± 22.38	6.76 ± 2.43	8.57 ± 4.58	301.72 ± 324.04
**CKD2**	127.75 ± 17.88	8.74 ± 1.67	12.50 ± 6.00	248.26 ± 265.58

Values are presented as mean ± SD. GS—German shepherds, BS—Belgian shepherds, SD—small dogs, y—years, CKD1—chronic kidney disease stage 1, CKD2—chronic kidney disease stage 2, Crea—serum creatinine, SDMA—symmetric dimethyl arginine, SUrom—serum uromodulin.

**Table 3:** Urinary markers in healthy dogs and dogs with kidney diseases.

	UPC RI: <0.2	UCrea (mmol/L)	UUrom (µg/mL)	UMC	UUrom/USG (µg/mL)
**GS < 2 y**	0.14 ± 0.03	18.44 ± 8.98	35.60 ± 24.22	18.10 ± 10.33	19.54 ± 11.61
**GS 2–6 y**	0.09 ± 0.04	19.45 ± 7.43	41.07 ± 37.22	19.85 ± 17.35	21.01 ± 19.11
**GS > 6 y**	0.12 ± 0.06	12.47 ± 6.98	35.68 ± 17.30	29.19 ± 20.72	25.18 ± 20.84
**BS**	0.14 ± 0.05	18.48 ± 6.90	19.23 ± 7.83	9.51 ± 2.65	12.9 ± 6.07
**SD**	0.15 ± 0.04	17.00 ± 4.89	45.48 ± 21.33	27.03 ± 17.32	33.5 ± 13.18
**CKD1**	0.10 ± 0.03	20.04 ± 11.69	29.40 ± 9.85	21.47 ± 24.41	26.4 ± 27.72
**CKD2**	0.27 ± 0.03	22.59 ± 6.90	29.71 ± 19.47	11.65 ± 5.99	14.84 ± 9.96

Values are presented as mean ± SD. GS—German shepherds, BS—Belgian shepherds, SD—small dogs, y—years, CKD1—chronic kidney disease stage 1, CKD2—chronic kidney disease stage 2, UPC—urinary protein-to-creatinine ratio, UCrea—urinary creatinine, UUrom—urinary uromodulin, UMC—urinary uromodulin-to-creatinine ratio, UUrom/USG—correction of uromodulin on urinary specific gravity.

## Data Availability

The original contributions presented in this study are included in the article.

## References

[1] Steubl D., Block M., Herbst V., Nockher W.A., Schlumberger W., Satanovskij R., Angermann S., Hasenau A.L., Stecher L., Heemann U. (2016). Plasma Uromodulin Correlates With Kidney Function and Identifies Early Stages in Chronic Kidney Disease Patients. Medicine.

[2] Devuyst O., Bochud M. (2015). Uromodulin, kidney function, cardiovascular disease, and mortality. Kidney Int..

[3] Schaeffer C., Devuyst O., Rampoldi L. (2021). Uromodulin: Roles in health and disease. Annu. Rev. Physiol..

[4] Devuyst O., Olinger E., Rampoldi L. (2017). Uromodulin: From physiology to rare and complex kidney disorders. Nat. Rev. Nephrol..

[5] Lv L., Wang J., Gao B., Wu L., Wang F., Cui Z., He K., Zhang L., Chen M., Zhao M.H. (2018). Serum uromodulin and progression of kidney disease in patients with chronic kidney disease. J. Transl. Med..

[6] Smirnov A.V., Khasun M., Kayukov I.G., Galkina O.V., Sipovski V.G., Parastaeva M.M., Bogdanova E.O. (2018). Serum uromodulin as an early biomarker of tubular atrophy and interstitial fibrosis in patients with glomerulopathies. Ter Arkh.

[7] Garimella P.S., Bartz T.M., Ix J.H., Chonchol M., Shlipak M.G., Devarajan P., Bennett M.R., Sarnak M.J. (2017). Urinary Uro-modulin and Risk of Urinary Tract Infections: The Cardiovascular Health Study. Am. J. Kidney Dis..

[8] Leiherer A., Muendlein A., Saely C., Brandtner E., Geiger K., Fraunberger P., Drexel H. (2018). The value of uromodulin as a new serum marker to predict decline in renal function. J. Hypertens..

[9] Risch L., Lhotta K., Meier D., Medina-Escobar P., Nydegger U., Risch M. (2014). The serum uromodulin level is associated with kidney function. Clin. Chem. Lab. Med..

[10] Ommen D. (2017). Uromodulin—A Protein with Many Clinical Implications—EUROIMMUNBlog. https://www.euroimmunblog.com/uromodulin-new-clinical-associations-html/.

[11] Ferlizza E., Isani G., Dondi F., Andreani G., Vasylyeva K., Bellei E., Almeida A.M., Matzapetakis M. (2020). Urinary proteome and metabolome in dogs (*Canis lupus familiaris*): The effect of chronic kidney disease. J. Proteomics.

[12] Perini-Perera S., Del-Ángel-Caraza J., Pérez-Sánchez A.P., Quijano-Hernández I.A., Recillas-Morales S. (2021). Evaluation of Chronic Kidney Disease Progression in Dogs With Therapeutic Management of Risk Factors. Front Vet. Sci..

[13] Coyne M., Szlosek D., Clements C., McCrann D., Olavessen L. (2020). Association between breed and renal biomarkers of glomerular filtration rate in dogs. Vet. Rec..

[14] O’Neill D.G., Coulson N.R., Church D.B., Brodbelt D.C. (2017). Demography and disorders of German Shepherd Dogs under primary veterinary care in the UK. Canine Genet. Epidemiol..

[15] International Renal Interest Society IRIS Staging of CKD. Modified 2023. http://www.iris-kidney.com/pdf/2_IRIS_Staging_of_CKD_2023.pdf.

[16] Youhanna S., Weber J., Beaujean V., Glaudemans B., Sobek J., Devuyst O. (2014). Determination of uromodulin in human urine: Influence of storage and processing. Nephrol. Dial. Transplant..

[17] Pruijm M., Ponte B., Ackermann D., Paccaud F., Guessous I., Ehret G., Pechère-Bertschi A., Vogt B., Mohaupt M.G., Martin P.Y. (2016). Associations of Urinary Uromodulin with Clinical Characteristics and Markers of Tubular Function in the General Population. Clin. J. Am. Soc. Nephrol..

[18] Troyanov S., Delmas-Frenette C., Bollée G., Youhanna S., Bruat V., Awadalla P., Devuyst O., Madore F. (2016). Clinical, Genetic, and Urinary Factors Associated with Uromodulin Excretion. Clin. J. Am. Soc. Nephrol..

[19] Shimmin L., Chang B., Hewett-Emmett D., Li W. (1993). Potential problems in estimating the male-to-female mutation rate ratio from DNA sequence data. J. Mol. Evol..

[20] Gluhovschi G., Gluhovschi A., Anastasiu D., Petrica L., Gluhovschi C., Velciov S. (2012). Chronic kidney disease and the involvement of estrogen hormones in its pathogenesis and progression. Rom. J. Intern. Med..

[21] Nanamatsu A., Micanovic R., Khan S., El-Achkar T.M., LaFavers K.A. (2023). Healthy Women Have Higher Systemic Uromodulin Levels: Identification of Uromodulin as an Estrogen Responsive Gene. Kidney360.

[22] Spinella G., Valentini S., Matarazzo M., Tidu L., Ferlizza E., Isani G., Andreani G. (2023). Effects of exercise on urinary biochemical parameters and proteins in a group of well-trained military working dogs. Vet. Q..

[23] Pivin E., Ponte B., de Seigneux S., Ackermann D., Guessous I., Ehret G., Pechère-Bertschi A., Olinger E., Mohaupt M., Vogt B. (2018). Uromodulin and Nephron Mass. Clin. J. Am. Soc. Nephrol..

[24] Hall J., Henegar J., Dwyer T., Liu J., da Silva A., Kuo J., Tallam L. (2004). Is obesity a major cause of chronic kidney disease?. Adv. Ren. Replace. Ther..

[25] Seo D., Yang Y., Hwang S.H., Jung J.H., Cho S., Choi G., Kim Y. (2022). Serum uromodulin in dogs with chronic kidney disease. J. Vet. Intern. Med..

[26] Prajczer S., Heidenreich U., Pfaller W., Kotanko P., Lhotta K., Jennings P. (2010). Evidence for a role of uromodulin in chronic kidney disease progression. Nephrol. Dial. Transplant..

[27] Raila J., Schweigert F., Kohn B. (2014). Relationship between urinary Tamm-Horsfall protein excretion and renal function in dogs with naturally occurring renal disease. Vet. Clin. Pathol..

[28] Chacar F., Kogika M., Sanches T.R., Caragelasco D., Martorelli C., Rodrigues C., Capcha J.M.C., Chew D., Andrade L. (2017). Urinary tamm-Horsfall protein, albumin, vitamin D-binding protein, and retinol-binding protein as early biomarkers of chronic kidney disease in dogs. Physiol. Rep..

[29] Będzichowska A., Jobs K., Kloc M., Bujnowska A., Kalicki B. (2021). The Assessment of the Usefulness of Selected Markers in the Diagnosis of Chronic Kidney Disease in Children. Biomark. Insights.

[30] Yazdani B., Delgado G.E., Scharnagl H., Krämer B.K., Drexel H., März W., Scherberich J.E., Leiherer A., Kleber M.E. (2021). Combined Use of Serum Uromodulin and eGFR to Estimate Mortality Risk. Front Med..

[31] Neely B., Bland A., Janech M. (2018). Proteomic Analysis of Urine from California Sea Lions (Zalophus californianus): A Re-source for Urinary Biomarker Discovery. J. Proteome Res..

[32] Karagiannidis A.G., Theodorakopoulou M.P., Pella E., Sarafidis P.A., Ortiz A. (2024). Uromodulin biology. Nephrol. Dial. Transplant.

[33] Iwasa N., Takashima S., Iwasa T., Kumazawa R., Nomura S., Asami S., Shimizu M., Kobatake Y., Nishii N. (2022). Effect of age, sex, and breed on serum cystatin C and creatinine concentrations in dogs. Vet. Res. Commun..

[34] Kuleš J., Bilić P., Beer Ljubić B., Gotić J., Crnogaj M., Brkljačić M., Mrljak V. (2018). Glomerular and tubular kidney damage markers in canine babesiosis caused by Babesia canis. Ticks Tick. Borne Dis..

